# Diosmetin attenuates oxidative stress-induced damage to lens epithelial cells via the mitogen-activated protein kinase (MAPK) pathway

**DOI:** 10.1080/21655979.2022.2068755

**Published:** 2022-04-28

**Authors:** Guanghai Guo, Jin Dong

**Affiliations:** a Department of Ophthalmology, Feicheng Hospital of Shandong Yiyang Health Group, Shandong, Feicheng, P.R. China; b Department of Clinical Laboratory, Feicheng Hospital of Shandong Yiyang Health Group, Shandong, Feicheng, P.R. China

**Keywords:** Diosmetin, oxidative stress, lens epithelial cell, MAPK pathway

## Abstract

Cataract is a global ophthalmic disease that blinds the eye, and oxidative stress is one of its primary causes. Apoptosis of lens epithelial cells (LECs) is considered the major cytological basis of many cataracts except congenital cataracts. The purpose of this study was to investigate whether diosmetin could reduce oxidative stress-induced damage to LECs, and explore its regulatory pathway. Lens epithelial cell line SRA01/04 was used as the object of study. Using ultraviolet B (UVB) and hydrogen peroxide (H_2_O_2_) as sources of oxidative stress, the protective effects of diosmetin at different concentrations on cells were investigated, including inhibition of proliferation, apoptosis, and oxidative stress. Molecular docking was then used to predict the target proteins and validation was performed at the cellular and protein levels. The oxidative stress of SRA01/04 was induced by UVB and H_2_O_2_, and inhibition of proliferation and apoptosis were observed. Here, diosmetin has a dose-dependent cell-protecting effect. This effect is achieved by targeting the MEK2 protein and inhibiting the MAPK signaling. In conclusion, diosmetin reduces H_2_O_2_- and UVB-induced inhibition of SRA01/04 proliferation and apoptosis by reducing oxidative stress-induced activation of the MAPK pathway.

## Graphical Abstract

**Figure:**
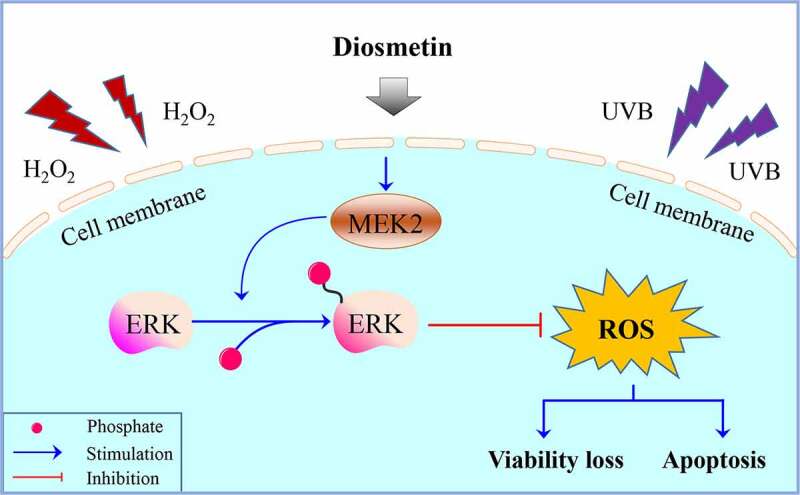

## Highlights

- Diosmetin promotes SRA01/04 cell proliferation;
- Diosmetin inhibits H_2_O_2_- and UVB-induced apoptosis of SRA01/04 cells;
- Diosmetin reduces oxidative stress induced by H_2_O_2_ and UVB;
- Diosmetin attenuates H_2_O_2_- and UVB-induced cell damage via regulating MEK2.

## Introduction

Cataract is the clouding of the lens caused by many reasons. It is a common senile disease and the world’s first eye disease. Surgery is still the most effective method of treating cataracts. Existing studies show many causes of cataracts. Hydrogen peroxide (H_2_O_2_) was significantly increased in the anterior chamber of most cataract patients [1]. Spector A et al. reported that oxidative stress occurs in the early stage of cataract development and the H_2_O_2_ level of cataract patients is 2 to 7 times the normal range [2]. The ROS concentrations in the anterior chamber and lens of cataract patients were significantly higher than in normal controls [3]. H_2_O_2_ induces apoptosis of lens epithelial cells (LECs) equivalent to the pathological changes in the eyes of cataract patients [4]. In the cataract process, oxidative stress first attacks the LECs.

LECs are the most metabolically active part of the lens. They can divide, proliferate, elongate at the equator, produce lens fibers, and maintain the metabolism of the entire lens. They play a crucial role in maintaining lens transparency [5]. Because of the importance of their anatomical location and physiological function, LECs are the primary target of oxidative stress and other injury factors. Li et al. found that oxidative damage can cause apoptosis of LECs, and cataracts appeared several hours after apoptosis, which gradually worsened over time, eventually leading to complete opacification. Apoptosis of LECs is considered the common cytological basis of all types of cataracts, except congenital cataracts [6]. Absence of epithelial cells in lenses from apoptosis-related death leads to cortical swelling, vacuolation, and subcapsular opacification, which eventually forms cataracts first in the equatorial region and then spreads to the entire lens [7]. So, protecting LECs from oxidative stress is the current idea of developing drugs to treat cataracts.

In traditional Chinese medicine (TCM), chrysanthemum purifies the liver, brightens the eyes, and is a key ingredient in TCM prescriptions for cataract treatment. Recent studies have shown that chrysanthemum extracts have anti-inflammatory, antioxidant, and anti-apoptotic abilities [8,9]. As an essential active ingredient in chrysanthemum, and diosmetin is a flavonoid compound that can regulate so many physiological activities. Its antioxidant and anti-apoptotic properties can protect tissues under various pathological conditions. For example, it was recently reported that diosmetin plays a cardioprotective role in myocardial ischemia injury in newborn rats by reducing oxidative stress and myocardial cell apoptosis [10]. Diosmetin has antioxidant, anti-inflammatory, and anti-apoptotic effects and can prevent endotoxin-induced acute liver failure in mice [11].

Based on the above research background, we speculated diosmetin might decrease the oxidative stress-induced damage of LECs. To test this hypothesis, we planned to use the human lens epithelial cell line SRA01/04 as a study object and induce oxidative stress of the cells by UVB radiation and H_2_O_2_ treatment. The protective effects of diosmetin on LECs were confirmed *in vitro*. The target protein was predicted and verified by molecular docking. Through the above experiments, we expected to confirm that diosmetin eases the damage of LECs caused by oxidative stress and explore its mechanism, providing theoretical support for a new drug therapy for cataracts.

## Materials and methods

### Cell culturing and treatments

Human LECs (SRA01/04; RCB1591, Riken BRC, Tsukuba, Japan) were cultured in DMEM/F-12 medium (Cat. No. 31331093, Gibco, USA) containing 10% fetal bovine serum (FBS; Cat. No. 10099141, Gibco, USA) and 1% penicillin/streptomycin (Cat. No. P1400, Solarbio, China) at 37°C with 5% CO_2_. When 60% confluence was reached, cells were treated with 200 μM H_2_O_2_ for 12 h or irradiated with UVB (2 W/m^2^) for 60 min and incubated for 12 h before the following experiments [12,13]. For UVB irradiation, cells in phosphate buffer saline (PBS) were irradiated from top to bottom with a UVB lamp (Photoelectric Instrument Factory, Beijing Normal University, Beijing, China) at a distance of 5 cm. The UVB light had a wavelength spectral distribution of 275–400 nm, with a peak at 310 nm. Diosmetin (Cat. No. A0927, Chengdu Must Biotechnology, China) was dissolved in dimethyl sulfoxide (DMSO) and further diluted in medium to different concentration levels (10, 20, and 40 μM).

### MTT assays

Cell proliferation was determined by MTT assays. The cells were seeded in a 96-well plate with 5 × 10^3^ cells per well. After 24 hours the cells were treated with H_2_O_2_ or UVB, and then 20 µL MTT (5 mg/mL; Cat. No. M1020, Solarbio, China) was added. Four hours later, the mixed medium was replaced with 150 µL DMSO (Cat. No. D8370, Solarbio, China). After the plate was agitated for 15 minutes, the optical density (OD) value of each well was measured using a fluorescence microplate reader at a wavelength of 490 nm.

### Cell apoptosis assays

Cell was assayed with the Annexin V-FITC Apoptosis Detection Kit (Cat. No. CA1020, Solarbio, China). Briefly, SRA01/04 cells at a concentration of 5 × 10^5^ cells/mL were collected and washed twice by PBS at 4°C, and then centrifuged at 300 g for 5 min at 4°C. The cells were then resuspended in 50  μL of binding buffer and incubated with the Annexin V-FITC and PI staining solution at room temperature for 10–15 min. Afterward, 250 μL of binding buffer was added to the mixture and cell apoptosis was measured by flow cytometry.

### Western blot assays

Western blot assay was used to detect protein expression levels in SRA01/04 cells. Proteins were extracted using RIPA total protein lysate (Cat. No. P0013C, Beyotime, China) and separated by electrophoresis on 10% SDS-PAGE, then blotted onto PVDF membranes (Cat. No. FFP39, Beyotime, China). After incubation with blocking solution (Cat. No. P0252, Beyotime, China), PVDF membranes were probed with anti-p53, anti-Bax, anti-Bcl-2, anti-p-p44/42, anti-p44/42, anti-p-JNK, anti-JNK, anti-p-p38, anti-p38, and anti-GAPDH antibodies (Cat. No. ab32389, ab32503, ab32124, ab278538, ab184699, ab124956, ab179461, ab178867, ab170099, ab9485, Abcam, USA, 1:1000) at 4°C overnight. After washing, the blots were incubated with an HRP-conjugated Goat Anti-Rabbit IgG H&L secondary antibody (Cat. No. ab6721, Abcam, USA, 1:2000) for 1 h and treated with the ECL Western Blot Reagent (Cat. No. P0018S, Beyotime, China) with a chemiluminescent imaging system (ChemiDoc MP, Bio-rad, USA).

### ROS detection

Reactive oxygen species (ROS) detection was performed using a ROS detection kit (Cat. No. CA1410, Solarbio, China). Briefly, the supernatant was discarded, and DCFH-DA (10 μM) diluted with DMEM/F-12 was added to the plate. The cells were then further cultured for 20 minutes, washed three times to remove DCFH-DA from the plate. The ROS content was determined by flow cytometry (BD Accuri™ C6 Plus, BD Biosciences, USA).

### Enzyme-linked immunosorbent assay (ELISA)

Superoxide dismutase (SOD), catalase (CAT), and glutathione (GSH) levels were measured in SRA01/04 cells using ELISA kits (Cat. No. BC0170, BC0205, and BC1175, Solarbio, China) according to the manufacturer’s instructions. OD values were measured in each well using a microplate reader (Multiskan FC, Thermo Scientific, USA).

### Target protein prediction and docking

The target protein of diosmetin was predicted using PharmMapper (http://www.lilab-ecust.cn/pharmmapper/) [14,15]. AutoDock Vina was then used to dock diosmetin to the predicted target protein to get minimal free energy [16,17], and Discovery Studio software was used for molecular interaction analysis.

### Statistical analysis

The researchers used the GraphPad Prism software (version 8.0) for mapping and statistical analysis. To analyze differences between groups, a one-way analysis of variance (ANOVA) followed by a Bonferroni post-hoc test was used. At p < 0.05, the data were considered significantly different. Image analysis was performed using the Fiji version of ImageJ [18,19].

## Results

### Diosmetin alleviates inhibition of SRA01/04 proliferation induced by H_2_O_2_ or UVB

**Figure 1.**
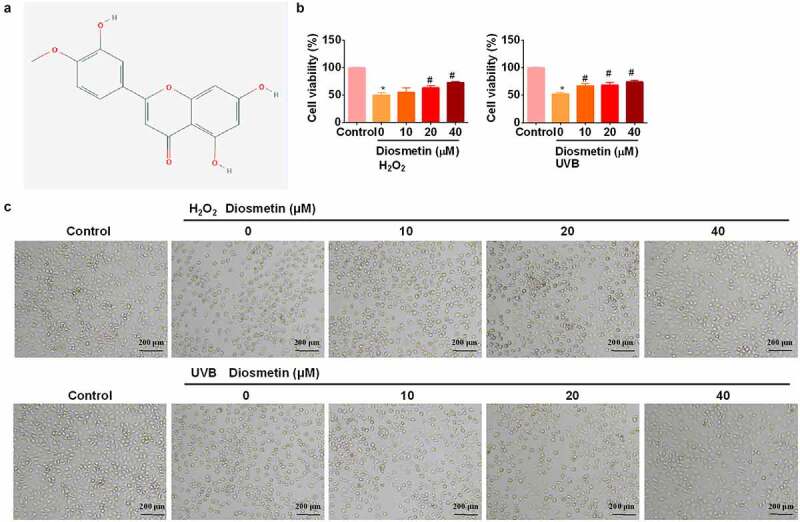
Diosmetin alleviates inhibition of SRA01/04 proliferation induced by H_2_O_2_ or UVB. (a) The 2D structure of diosmetin; (b) MTT test results of SRA01/04 cells under different conditions; (c) Images captured by the bright filed microscope to show the morphological changes. *P < 0.05 compared with control group, #P < 0.05 compared with 0 μM diosmetin group.

First, the effects of diosmetin on H_2_O_2_- and UVB-induced loss of viability of LECs were examined. The chemical structures of diosmetin are shown in Figure 1a. Based on the results of previous studies [12] we treated cells with H_2_O_2_ or UVB light. Cells were treated with 200 μM H_2_O_2_ for 12 h or UVB (2 W/m^2^) irradiation for 60 min and an MTT assay was performed. As shown in Figure 1b, proliferation of SRA01/04 cells was significantly inhibited (*p* < 0.05) by H_2_O_2_ or UVB. Adding different doses of diosmetin to H_2_O_2_- or UVB-treated cells effectively alleviated the inhibition of proliferation in a dose-dependent manner (*p*< 0.05). Cell morphological changes in SRA01/04 cells were monitored by microscopy. Compared to the untreated control group, the cells exposed to H_2_O_2_ and UVB appeared to be round, shrank and many floating cells were observed (Figure 1c). However, the H_2_O_2_- and UVB-induced morphology changes of SRA01/04 cells were restored by diosmetin treatment in a dose-dependent manner.

### Diosmetin reduced H_2_O_2_- and UVB-induced apoptosis of SRA01/04 cells

**Figure 2.**
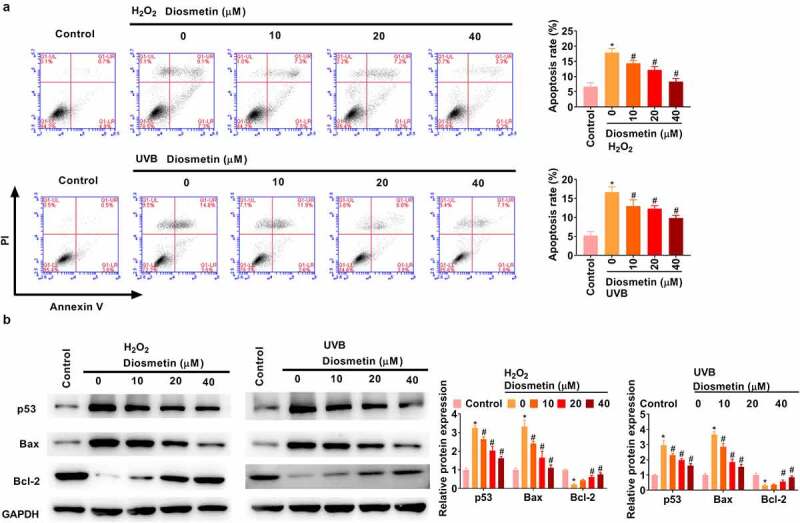
Diosmetin reduced H_2_O_2_- and UVB-induced apoptosis of SRA01/04 cells. (a) Cell apoptosis was detected by flow cytometry; (b) The expression of apoptosis-related proteins was detected by western blotting assay. *P < 0.05 compared with control group, #P < 0.05 compared with 0 μM diosmetin group.

The effects of diosmetin on H_2_O_2_- and UVB-induced apoptosis were then examined. As shown in Figure 2a, H_2_O_2_ and UVB light could induce apoptosis of SRA01/04 cells, while diosmetin could effectively alleviate apoptosis, and its cell protective effect was dose dependent (*p*< 0.05). Subsequently, changes in the expression levels of the apoptosis-related proteins p53, Bax, and Bcl-2 were detected under different conditions. As shown in Figure 2b, H_2_O_2_ or UVB light significantly enhanced the expression levels of p53 and Bax and significantly down-regulated the expression levels of Bcl-2, while diosmetin at different doses could effectively ameliorate the changes in protein expression levels induced by H_2_O_2_ or UVB dose-dependently (*p*< 0.05).

### Diosmetin reduces oxidative stress induced by H_2_O_2_ and UVB light

**Figure 3.**
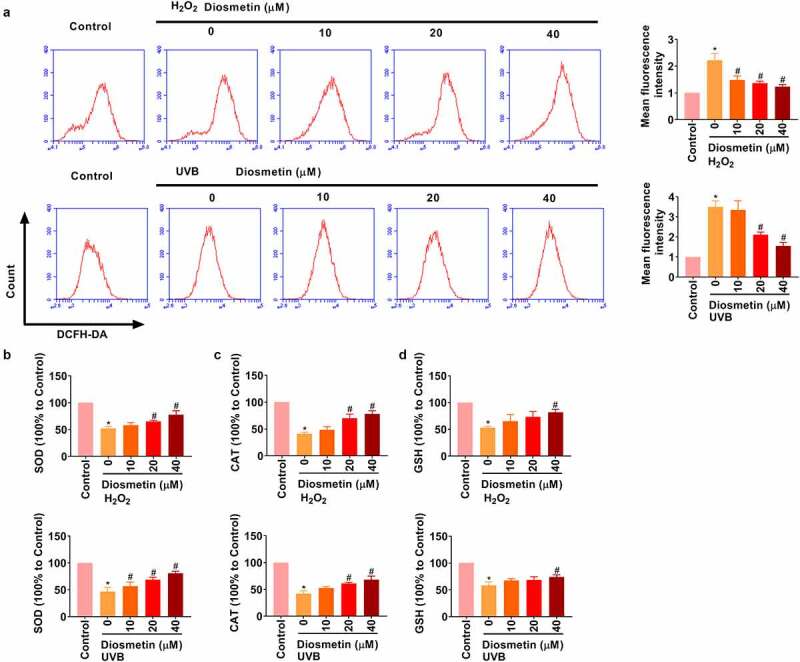
Diosmetin reduces oxidative stress induced by H_2_O_2_ and UVB. (a) The detection of the production of ROS in cells under different conditions; The changes of (b) SOD, (c) CAT, and (d) GSH were detected by ELISA under different conditions. *P < 0.05 compared with control group, #P < 0.05 compared with 0 μM diosmetin group.

Next, the effects of diosmetin on H_2_O_2_ and UVB-induced oxidative stress were demonstrated. Under stimulation of H_2_O_2_ and UVB, intracellular ROS was detected by flow cytometry. As results in Figure 3a show, H_2_O_2_ or UVB light could induce the increase in ROS level in SRA01/04 cells, and diosmetin could effectively reduce ROS level in a dose-dependent manner (*p*< 0.05). ELISA showed that H_2_O_2_ or UVB significantly repressed SOD, CAT, and GSH levels, while different doses of diosmetin were effective in alleviating H_2_O_2_- or UVB-induced changes in protein levels in a dose-dependent manner, as shown in Figure 3b–d (*p*< 0.05).

### Diosmetin attenuates H_2_O_2_- and UVB-induced activation of MAPK signaling pathways

**Figure 4.**
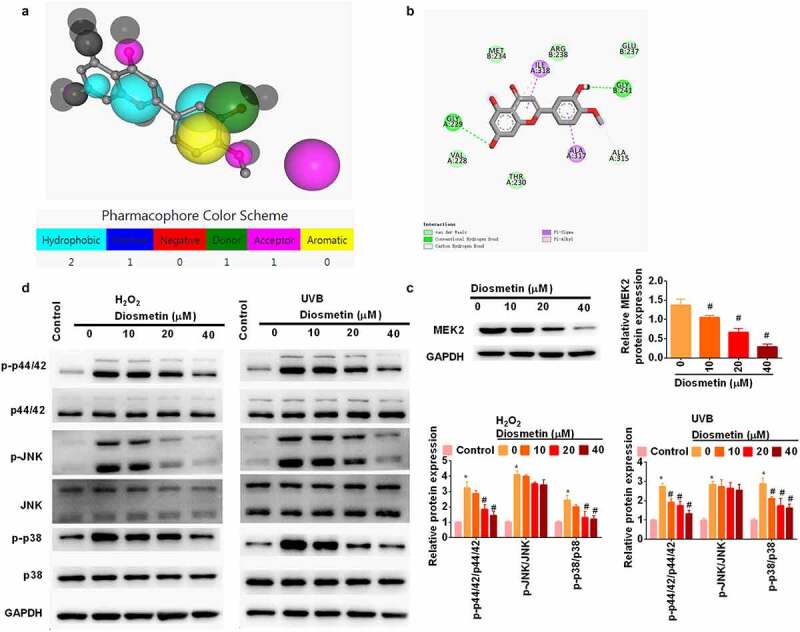
Diosmetin attenuates H_2_O_2_- and UVB-induced activation of MAPK signaling pathways. (a) Molecular and pharmacophore models of diosmetin binding MEK2; (b) Molecular interaction analysis of diosmetin and MEK2; (c) Western blot was performed to detect the expression of MEK2 in diosmetin-treated cells; (d) Western blot was used to detect the phosphorylation of MAPK pathway related proteins. *P < 0.05 compared with control group, #P < 0.05 compared with 0 μM diosmetin group.

To further understand how diosmetin exerts its protective effect, PharmMapper was used to predict the target protein (http://www.lilab-ecust.cn/pharmmapper/) [14,15,20] of diosmetin. The results showed that mitogen-activated protein kinase 2 (MEK2) may be the target protein of diosmetin. The Molecule and Pharmacophore Model are shown in Figure 4a. To further confirm this prediction, Autodock Vina was used for molecular docking and nine conformations were obtained, among which the lowest binding energy was −6.8 Kcal/mol. Then, discovery Studio was used for molecular interaction analysis (Figure 4b). MEK2 is a dual protein tyrosine/threonine kinase found in the Ras/Raf/MEK/ERK signaling pathway of mitogen-activated protein kinase (MAPK) [21]. Researchers of this study further used western blotting to identify the direct regulation of diosmetin on MEK2 expression. Diosmetin significantly reduced the protein levels of MEK2 in a dose-dependent manner (*p*< 0.05, Figure 4c). In addition, H_2_O_2_ and UVB significantly (*p* < 0.05, Figure 4d) phosphorylated MAPK-related proteins (p44/42, JNK, and p38). Treatment with diosmetin significantly reduced phosphorylation of p44/42 and p38 (*p*< 0.05) rather than JNK (*p*> 0.05).

### The combination of diosmetin and MEK inhibitor PD98059 reduces H_2_O_2_- and UVB-induced proliferation inhibition and apoptosis

**Figure 5.**
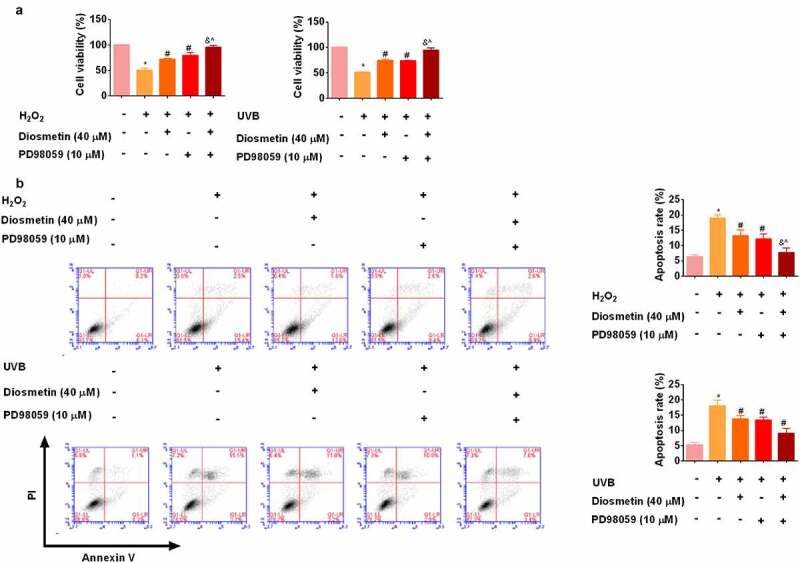
The combination of diosmetin and MEK inhibitor PD98059 reduces H_2_O_2_- and UVB-induced proliferation inhibition and apoptosis. (a) MTT test results of SRA01/04 cells under different conditions; (b) Cell apoptosis was detected by flow cytometry. * P < 0.05 compared with control group; # P < 0.05 compared with 0 μM diosmetin, 0 μM PD98059 group; & P < 0.05 compared with 40 μM diosmetin, 0 μM PD98059 group; ^P < 0.05 compared with 0 μM diosmetin, 10 μM PD98059 group.

To determine whether diosmetin worked by modulating MEK2, a MEK inhibitor PD98059 was then administered to cells. As shown in Figure 5a-b, MTT results showed that diosmetin or PD98059 eased proliferation inhibition and apoptosis of cells under oxidative stress (*p* < 0.05). The combination of diosmetin and PD98059 enhanced the remission ability (*p* < 0.05). These results showed that inhibition of MAPK pathway can protect cells under oxidative stress.

## Discussion

This study confirmed that diosmetin reduces the damage to LECs induced by oxidative stress. The results showed diosmetin reduced H_2_O_2_- and UVB-induced proliferation inhibition and apoptosis of SRA01/04 cells by reducing oxidative stress-induced activation of the MAPK pathway. This study provides theoretical support for the new prophylactic and therapeutic methods of drug therapy for cataracts.

Currently, several bioactive compounds have been found to possess excellent antioxidant properties and protective effects against eye diseases [22]. For instance, Lycium barbarum polysaccharide delivered by nano-selenium protected LECs against UVB damage [23]. Epigallocatechin gallate prevented UVB-induced oxidative stress and inhibited apoptosis-related death of LECs [24]. Ganoderic acid A protected LECs against UVB irradiation and delayed lens opacity [25]. Paeoniflorin inhibited epithelial–mesenchymal transformation and oxidative damage to LECs and exhibited benefits in preventing diabetic cataracts [26]. Diosmetin has presently been reported as a potential agent for improving eye function by restoring oxidative stress and DNA injury in the retinal pigment epithelium layer [27]. We showed that diosmetin is a potential candidate to prevent cataracts. Diosmetin treatment significantly prevented oxidative stress-induced LEC damage.

After a series of verifications of the protective effects of diosmetin on SRA01/04 cells, PharmMapper was used to predict the target protein of diosmetin. PharmMapper applies Cavity to detect the potential-binding sites on the surface of a protein structure and ranks them according to the Druggability scores [28]. Molecular docking by AutoDock furtherly confirmed the binding relationship and strength of diosmetin and MEK2. Several software and algorithms helped improve the accuracy of the prediction. The subsequent western blotting findings confirmed the previous experimental results.

Besides inhibiting cell damage induced by oxidative stress, several studies have shown that diosmetin can also play a protective role in other ways. For example, diosmetin also has an iron-chelating ability, protecting cells by chelating excessive iron ions in cells [29]. Diosmetin increases vascular tone by inhibiting dopamine and serotonin uptake [30] and inhibits the activation of carcinogens by directly inhibiting cytochrome P450 1A1 (CYP1A1) [31].

In the last part of the study, we explored the synergistic enhancement of diosmetin combined with the MEK inhibitor PD98059. PD98059 inhibited MEK1 and MEK2 with an IC50 value of 4 µM for MEK1 and 50 µM for MEK2 [32–34]. MAPK is a phosphorylated serine/threonine protein kinase with high cytoplasmic content [35]. MAPK signaling pathway is an important intracellular signal transduction pathway that regulates cell reproduction, growth, apoptosis, differentiation, and other biological activities, but can also be activated by different extracellular stimuli [36–38]. The MAPK signaling pathway plays many significant roles in eye diseases. For example, studies have shown that exogenous nitric oxide (NO) can activate the MAPK pathway to regulate the expression of inflammatory cytokines on the ocular surface and enhance corneal wound healing [39]. In contrast, the MAPK/ERK pathway is involved in epidermal growth factor (EGF)-mediated up-regulation of Ca (2+)-K(+)-K(+) channel in the mouse corneal alkali combustion model, and reduces EGF-induced neovascularization [40]. In the current study, MEK2 was a direct target of diosmetin, and diosmetin protected LECs against oxidative stress damage, possibly by regulating MAPK signaling.

## Conclusion

Taken together, by targeting MEK2 and reducing oxidative stress-induced MAPK pathway activation, diosmetin helps to protect lens epithelial cells against H_2_O_2_ and UVB-induced damage, suggesting diosmetin as a potential candidate for cataract treatment.

## Supplementary Material

Supplemental Material

## Data Availability

The datasets used and analyzed during the current study are available from the corresponding author (E-mail: lilill21@163.com) on reasonable request.
