# Supplementary material for: Diosmetin attenuates oxidative stress-induced damage to lens epithelial cells via the mitogen-activated protein kinase (MAPK) pathway
Source: Bioengineered. 2022 Apr 28;13(4):11072–81. doi: 10.1080/21655979.2022.2068755 (PMC9208454; doi:10.1080/21655979.2022.2068755)
Supplement: Supplemental Material [file KBIE_A_2068755_SM7284.zip › supplementary/downloadFromZipFile.pdf]

TOPEDIT

TOPEDIT SCIENTIFIC EDITING

CERTIFICATE OF ENGLISH EDITING

This certificate confirms that the manuscript listed below was edited by one or more expert English editors with doctoral degree. The following issues were corrected: grammar, spelling, punctuation, sentence structure, and phrasing. Journal editors can contact us at info@topeditsci.com if you have any concerns.

Manuscript title

Diosmetin attenuates oxidative stress-induce d lens epithelial cell damage by mitogen-acti vated protein kinase (MAPK) pathway

Date issued

03/31/2022

Certificate number

CN 7568-05-0331-01

TopEdit specializes in comprehensive preparation of scientific manuscripts that are targeted for publication in English language journals. We provide editing, technical translation, formatting, and illustration services for technical manuscripts, grant proposals, conference presentations and posters, and professional school application essays and other scientific materials. All orders are edited by one or more editors who hold advanced Ph.D. degrees and are either natively proficient in English or native speakers.

https://www.topeditsci.com
