# Supplementary material for: Diosmetin attenuates oxidative stress-induced damage to lens epithelial cells via the mitogen-activated protein kinase (MAPK) pathway
Source: Bioengineered. 2022 Apr 28;13(4):11072–81. doi: 10.1080/21655979.2022.2068755 (PMC9208454; doi:10.1080/21655979.2022.2068755)
Supplement: Supplemental Material [file KBIE_A_2068755_SM7284.zip › supplementary/Original Western Blots.docx]

**Fig2B**

**H_2_O_2_**

**P53**

**



**

**BAX**

**



**

**Bcl-2**

**



**

**GAPDH**

**



**

**Fig2B**

**UVB**

**P53**

**



**

**BAX**

**



**

**BCL-2**

**



**

**GAPDH**

**



**

**Fig4C**

**MEK2**

**



**

**GAPDH**

**



**

**Fig4D**

**H_2_O_2_**

**p-p44/42**

**



**

**p44/42**

**



**

**p-JNK**

**



**

**JNK**

**



**

**p-p38**

**



**

**p38**

**



**

**GAPDH**

**



**

**Fig4D**

**UVB**

**p-p44/42**

**



**

**P44/42**

**



**

**p-JNK**

**



**

**JNK**

**



**

**p-p38**

**



**

**P38**

**



**

**GAPDH**

**



**
